# Engineering Translation in Mammalian Cell Factories to Increase Protein Yield: The Unexpected Use of Long Non-Coding SINEUP RNAs

**DOI:** 10.1016/j.csbj.2016.10.004

**Published:** 2016-10-27

**Authors:** Silvia Zucchelli, Laura Patrucco, Francesca Persichetti, Stefano Gustincich, Diego Cotella

**Affiliations:** a Department of Health Sciences, Università del Piemonte Orientale, Novara, Italy; b Area of Neuroscience, SISSA, Trieste, Italy; c Department of Neuroscience and Brain Technologies, Italian Institute of Technology (IIT), Genova, Italy

**Keywords:** CHO, Chinese hamster ovary, ER, Endoplasmic reticulum, lncRNA, long non-coding RNA, MAb, monoclonal antibody, SINE, short interspersed nuclear element, SME, small and medium-sized enterprise, SP, Signal peptide, Cell factory, Recombinant protein, Protein translation, Signal peptide, lncRNA, SINEUP

## Abstract

Mammalian cells are an indispensable tool for the production of recombinant proteins in contexts where function depends on post-translational modifications. Among them, Chinese Hamster Ovary (CHO) cells are the primary factories for the production of therapeutic proteins, including monoclonal antibodies (MAbs). To improve expression and stability, several methodologies have been adopted, including methods based on media formulation, selective pressure and cell- or vector engineering. This review presents current approaches aimed at improving mammalian cell factories that are based on the enhancement of translation. Among well-established techniques (codon optimization and improvement of mRNA secondary structure), we describe SINEUPs, a family of antisense long non-coding RNAs that are able to increase translation of partially overlapping protein-coding mRNAs. By exploiting their modular structure, SINEUP molecules can be designed to target virtually any mRNA of interest, and thus to increase the production of secreted proteins. Thus, synthetic SINEUPs represent a new versatile tool to improve the production of secreted proteins in biomanufacturing processes.

## 1 Introduction

### 1.1 Overview on Mammalian Cell Factories

Recombinant proteins are invaluable resources for basic research and for biotechnological applications. They can be produced in several different expression systems, but mammalian cells are the best choice when post-translational processing (*e.g.* glycosylation) is required for their function. This is crucial for proteins of therapeutic interest. In the past 20 years, over two hundreds of recombinant proteins have been approved by the European Medicine Agency (EMA) [1]. Among these proteins, monoclonal antibodies (MAbs) represent the biotech industry's fastest growing sector [2], [3], [4], [5], [6]. Chinese Hamster Ovary (CHO) cells are the leading factories for the production of recombinant MAbs, as they have superseded “classical” MAbs produced in mice [7], [8]. CHO cells are safe and robust hosts in which high productivity can be achieved *via* insertion of multiple copies of the transgenes [9]. In addition, CHO cells can be easily adapted to grow in suspension, in serum-free conditions and at high cell densities [10]. However, CHO cells possess also some unwanted traits, such as a relevant genome instability; they are also inclined to epigenetic silencing [11], [12]. Since undesired traits affect clone productivity (in terms of both quantity and quality), different strategies have been adopted to attenuate these disadvantages. Some of them regard the design of the expression vector and, for example, make use of inducible promoters and/or epigenetic regulators to increase and prolong transgene expression while decreasing toxicity of the expressed recombinant protein [13], [14], [15], [16]. Others approaches aim at manipulating pathways through cell engineering, in order to improve stress resistance, cell viability or to achieve better glycosylation profiles [7], [17]. Despite much progress has been made in this field, clonal variability and instability are still important issues that need to be addressed, particularly when production on large scales (1000's liters) is required. Though it is certain that CHO cells will continue to be used and developed for the production of biologics, the pressure for generating more complex proteins has led to the further development of novel cell lines. Of particular interest are cell lines of human origin (*e.g.* HEK cells) that are expected to become the platforms of the future [4], [8], [18].

### 1.2 The Need for Further Advancements

**Fig. 1:**
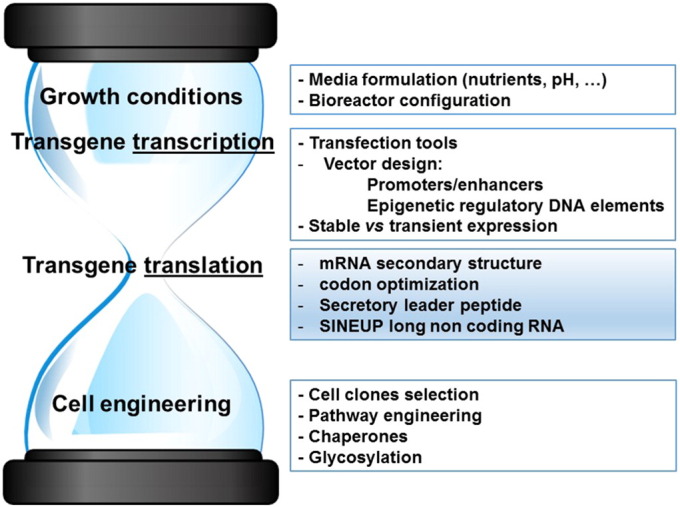
Summary of strategies adopted to optimize mammalian cell factories. The optimization of translation has been identified as a bottleneck among the several strategies to increase the production of recombinant proteins. It therefore represents a key issue that needs to be addressed to optimize mammalian cell factories.

The past few years have witnessed a countless development of strategies to improve the productivity of mammalian cell factories (summarized in Fig. 1). Indeed, protein yields are currently higher than ever, and it is now the norm to achieve multiple grams of recombinant protein per liter of culture media [19], [20]. Moreover, stable producer clones can now be generated within few weeks. However, therapies based on bio-therapeutics are still dozen of times more expensive than therapies based on small-molecule therapeutics [21], [22], [23]. As manufacturers attempt to reduce the size of production batches still maintaining them economically profitable, mammalian cells factories are propelled to their limits [24]. Such endeavors are necessary to sustain the development of personalized approaches to medicine, as a result of the progressive shift toward novel classes of MAb-based therapeutics [25]. Despite new technologies have contributed a considerable advance, expression levels are often too low to be economically rewarding.

Engineered CHO cells have been generated to enhance protein production at industrial scale. This has been made possible, recently, by the blast of *omics* data, which have improved our understanding of CHO biology [26], [27], [28], [29], [30]. In addition to this, CRISPR/Cas9 technology has been adopted to further dissect CHO biological determinants to productivity and to genome-engineer cells toward the development of next generation factories [31]. Nevertheless, the industry still needs a better understanding of the implications of new omics information.

We do expect that engineering cells at the level of transcription, translation and the secretory pathways would have an additive effect on productivity. Moreover, with the progress of systems biology, it will be possible to manipulate cells to introduce entire new molecular pathways (*e.g.* human-like glycosylation) [17], [29]. The rational engineering of such robust and high-performing cells for specific applications can lead to a catalog of different cell lines, each optimized to tackle specific targets.

In summary, any biotechnological improvement, even small, that can increase either the efficiency of protein synthesis or the quality of post-translational modifications is still very welcomed, and it is of potential interest for a number of biotech SMEs with different finalities.

## 2 Focus on the Enhancement of Translation

### 2.1 mRNA Secondary Structure and Codon Optimization

As most endogenous eukaryotic proteins, recombinant proteins are usually expressed through cap-dependent, linear scanning mechanism of mRNA translation. This is a tightly regulated cellular mechanism, which consists of four main steps: initiation, elongation, termination and ribosome recycling (reviewed in [32]).

The initiation phase, in particular, determines the efficiency of mRNA translation, and thus represents the main rate-limiting step (reviewed in [33], [34], [35], [36], [37], [38]). In fact, whereas a relatively small number of dedicated factors is necessary to support the elongation and termination phases, the machinery required to initiate translation in eukaryotes is composed by more than 25 proteins [35]. In addition to this, translation of many mRNAs can be initiated by mechanisms that divert from the “canonical” pathway (mechanisms of CAP-independent translation not be discussed here; for references please refer to reviews [39], [40], [41]). However, they are generally not utilized in cells producing high quantities of recombinant proteins.

In the cap-dependent mechanism of mRNA translation, the cap-binding complex eIF4F (composed of the initiation factors eIF4E, eIF4G, and the RNA helicase eIF4A) binds the 7-methylguanosine cap (m7GpppG) at the 5′ of the mRNA, and then recruits the 40S ribosomal subunit as a 43S pre-initiation complex. The latter is composed by the 40S subunit, the initiation factors eIF3, eIF1, eIF1A, eIF5, and the methionyl-initiator tRNA (Met-tRNA_i_), in a pre-assembled ternary complex with GTP–bound form of eIF2. These factors serve to bring the 40S subunit to the 5′ end of the mRNA and load the mRNA onto the 40S ribosome. Then, the 40S subunit scans the mRNA in a 5′ to 3′ direction until the AUG start codon is recognized. Upon AUG recognition, GTP is hydrolysed, eIF1 is released, and the 40S subunit undergoes a conformational change that grips the mRNA and prevents further scanning. Lastly, the 60S subunit joins facilitated by eIF5B, and GTP hydrolysis triggers release of eIF1A and eIF5B to form a fully competent 80S ribosome.

The sequence encompassing the start AUG (the “Kozak” sequence) is crucial in helping the scanning ribosomal subunit to accomplish the proper codon-anticodon recognition. The optimal Kozak sequence is evolutionarily conserved, and the consensus for higher vertebrates is CC(R)CC**AUG**G. The purine (R) at position − 3 and the G at + 4 are the most crucial nucleotides (reviewed in [42]).

Ribosomal scanning has to face some structural obstacles, since RNA molecules naturally tend to form secondary structures, and those located in the 5′ UTRs of mammalian mRNA transcripts may affect translation efficiency.

The role of RNA structure in mRNA translation has been extensively investigated for more than 40 years. Those studies have been recently propelled with the advent of novel tools, *i.e.* SHAPE [43], [44] and CIRS-seq [45] that allow the systematic, whole-transcriptome analysis of RNA secondary structures; however, most of our current knowledge comes from Kozak's pioneering studies on the scanning model of mRNA translation (reviewed in [46]).

**Fig. 2:**
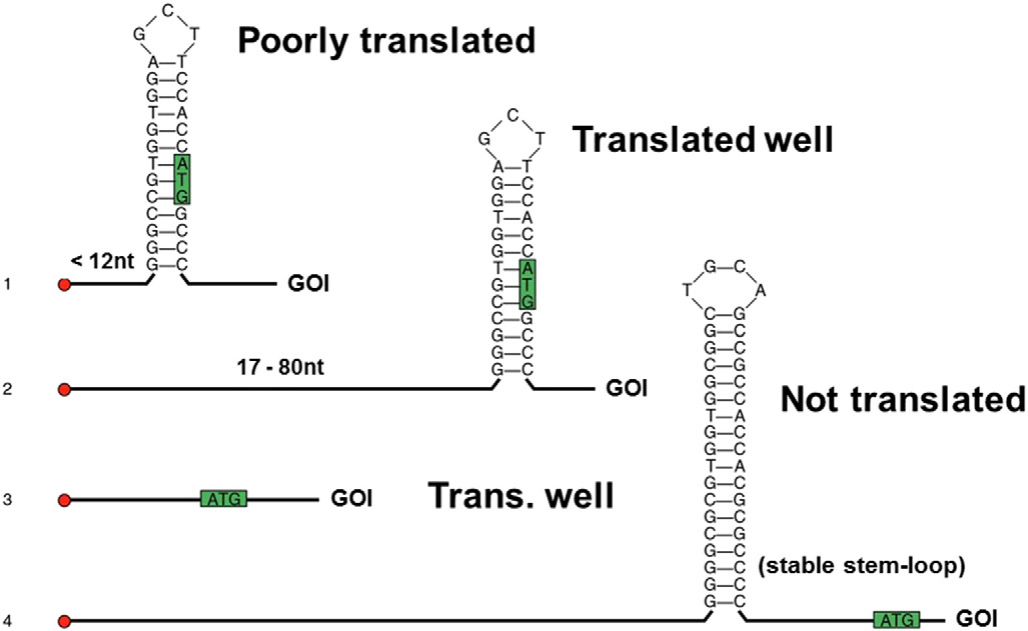
The mRNA secondary structure in the 5′ UTR and surrounding the starting AUG is a key determinant of translation efficiency. (1) The sAUG incorporated in a hairpin structure close to the 5′ end will result in lower levels of translation. (2) The same structure as above, but in longer 5′ UTR sequences, will not affect significantly the translation efficiency. (3) A 5′ UTR devoid of secondary structures will be translated well. (4) A stable stem-loop near the starting AUG will block the ribosome scanning, preventing translational start. GOI: Gene of Interest. Adapted from data presented in ref. [47].

In general, the presence of stable secondary structures in the mRNA at 5′ UTR exerts a negative effect on the translation rate (summarized in Fig. 2). In particular, a stable stem-loop near the (m7GpppG) cap will reduce the efficiency of translation by preventing the access of eIF4F [47], [48], [49]. Similarly, a stem-loop in proximity of the start AUG will impede translation by interfering with the formation of the pre-initiation complex [46], [50], [51]. As the chances to fold into secondary structure increases with the length of RNA, this is probably one of the reasons why mammalian 5′ UTRs are usually short, in general between 100 and 200 nucleotides in length [52]. Although it is well documented that mRNA secondary structures are important to regulate the expression of endogenous genes (reviewed in [53]), from the point of view of cell factories they represent an obstacle to fully exploit the translation potential, and they should be removed. Ideally, the optimal 5′ UTR for highly efficient translation of recombinant protein should be devoid of any secondary structure. In addition to this, it should be also devoid of extra-AUG codons and near-cognate triplets in an optimum sequence context, to preclude potential translation of an upstream open reading frame (uORF) (reviewed in [38], [54]).

The 3′ UTRs of mRNAs also play a role in the regulation of the initiation phase of translation (reviewed in [55], [56]). In fact, after binding the poly(A) tail, the poly(A)-binding proteins (PABPs) interact with the eIF4G, thus increasing the affinity between eIF4E and the (m7GpppG) cap [50]. These protein–protein interactions cause the mRNA to adopt a pseudo-circular structure, bringing the mRNA head in close proximity to its tail, enabling the ribosome to restart translation more promptly, thereby determining an increase in the efficiency of translation (reviewed in [36], [57]).

The rate of elongation also influences translation efficiency. Elongation rate is determined, at least in part, by the efficiency of codon-anticodon recognition. In genomes, rarely used codons cause a pause in translation due to the low concentration of the corresponding aminoacyl-tRNA [58]. Several studies have shown that the production of recombinant proteins in heterologous systems may improve dramatically if the codon usage correlates with the codon bias, because of increased translation rate [59], [60], [61] and mRNA stability [62]. These findings have led to codon usage optimization strategies adjusted to the specific organism selected as cell factories (bacteria, yeast or mammalian cells).

However, such manipulation is not without drawbacks, as we know that codon bias is the result of a precise natural selection, In fact, recently, it has been demonstrated that frequently used codons accelerate elongation, while non-preferred codons slow it down, and altering the codon usage influences the local translational dynamics [63]. As a result of the evolutionary adaptation, the changes of translation elongation rates on mRNAs are adapted to protein structures to facilitate co-translational folding, suggesting a codon usage “code” for protein structure [63], [64], [65]; altering this code may negatively affect the functionality of the encoded protein.

Altogether, these findings inspired the development of several bioinformatics tools for the comprehensive, multiparametric optimization of translation products (Table 1 summarize only few of them). Progress in the development of tools for gene optimisation combined with *de novo* gene synthesis allow rapid and efficient construction of synthetic genes individually fitted to specific biotechnological needs. Previously, gene optimization was mainly performed by empirical site-directed mutagenesis of a DNA template [60], [66], [67]. With these novel tools it is now possible, following *in silico* sequence-optimization, to rapidly synthesize full-length genes based on the available DNA [68], [69], [70] or protein sequences [71], [72]. It is even possible to synthesize artificial genes with novel properties [73], [74], [75]. The classical example is insulin, the first recombinant protein approved for therapeutic use [76]. The amino acid sequence of “first generation” recombinant insulin is identical to native human insulin. With *de novo* gene synthesis it has been possible to produce insulin analogs displaying altered amino acid sequences aiming at improving their performances (the “second generation” insulins). To date, several such insulin analogs have been engineered to own either an accelerated (fast-acting) or prolonged duration of action (slow-acting) [77].

Chemical synthesis of long polynucleotides is now affordable and guarantees easy access to virtually any gene of interest, including those that are difficult to clone by classical PCR-based methods or have been inaccurately deposited in clone repositories.

### 2.2 Improving the Secretory Leader Sequence

Most recombinant proteins produced in mammalian cell factories are expressed in a secretable format [78], [79]. This is achieved by adding a signal peptide (SP), an amino acid sequence 5–30 residues in length, at the N-terminus of the protein of interest [80]. While still being synthesized on the ribosome, nascent poplypetides are recognized by the signal recognition particle (SRP) and addressed to the ER [81].

The translocation of secretory proteins into the lumen of the ER represents a bottleneck within the secretory pathway and thus depicts a key issue that needs to be addressed to exploit the full potential of mammalian cell factories. The appropriate selection of a SP can have important consequences on protein overexpression, with some authors reporting levels of expression increased by several-fold [82], [83], [84]. Studies have shown that, despite their heterogeneity, many SPs are functionally interchangeable even between different species [85]. Indeed, most SPs share three structurally conserved regions: an N-terminal polar region (N-region), rich in positively charged amino acids; a central hydrophobic region (H-region) composed of about 7–8 hydrophobic amino acids; and a C-terminal region (C-region) that includes the SP cleavage site [86], [87].

Different SPs can deeply impact protein secretion [82]. These observations should be taken in consideration when aiming at producing maximal amounts of recombinant proteins in mammalian cells. Many groups have demonstrated that protein production can be empowered using alternative SPs [70], [82], [85], [88], [89], [90]. Logically, the optimal choice for signal sequence may be the proteins native SP, though testing a small panel of commonly utilized signal sequences may be desirable. Several efficient and well-described signal sequences have been reported, including IL-2, IL-6 CD5, Immunoglobulins (Ig), trypsinogen, serum albumin, prolactin and elastin [8], [82], [83], [91], [92]. While some SP showed a broad skill in promoting protein secretion, others are more protein specific [82]. Thus, empirical trials may be needed to find the best SP suited for the protein of interest, in particular if the expression levels are low. A good example is the recent work published by Zhiwei Song and colleagues [70]. In this works, they generated a database of SPs from a large number of human Ig heavy chain (HC) and kappa light chain (LC), and analyzed for their impacts on the production of 5-top selling antibody therapeutics (Herceptin, Avastin, Remicade, Rituxan, and Humira). Interesting to note, the cDNA clones of those antibodies where chemically synthesized starting from DNA sequence information publicly available. Following this approach, it was possible to engineer the SP for Rituxan to achieve a 2-fold yield compared to its native SP.

A plethora of biological data on the structure/function relationship of SP are available, and they can now be exploited to develop bioinformatics tools to determine cleavage sites and the expression localization of various SPs (SignalP, TargetP, and PSORT [93], [94], [95]) and for the *in silico* design of artificial SPs. As an example, UTR-Tailortech allows the rational design of SP libraries randomized at chosen codon positions [84]. This tool was developed by comparing the success of individual SPs with their amino acid composition and has allowed to predict with respect to which amino acid in which positions can have a decisive influence on protein synthesis or secretion [84]. In contrast to a traditional random approach, which would result in extremely large libraries difficult to manage, libraries generated with UTR-Tailortech are substantially smaller while simultaneously being enriched for good candidates.

This increases the chances of finding “the needle in the haystack”. When combined with high-throughput screening technologies, a tailored SP for any specific protein (including difficult-to-express proteins) can be quickly defined [84].

### 2.3 Exploiting SINEUP Non-Coding RNAs to Improve the Translation of Recombinant Proteins

Translation improvement still needs to be further explored and incorporated into the production pipeline. As described above, a line of intervention is focused on the optimization of the mRNA sequence itself, either at the level of coding sequence and codon usage or at the 5′ and 3′ UTR sequences. Additional strategies are currently being developed to modulate translation with *trans*-acting, gene-specific regulatory long non-coding RNAs (lncRNAs).

**Fig. 3:**
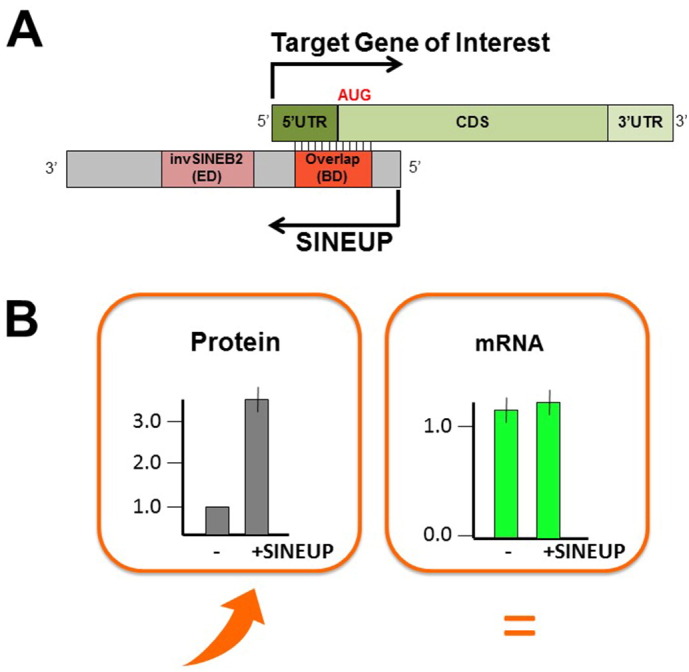
SINEUP modular structure and principle of action. A) SINEUPs are antisense lncRNAs that activate translation. SINEUPs contain two functional domains: the Binding Domain (BD) provides target specificity; the Effector Domain (ED) provides activation of protein synthesis. 5′ to 3′ orientation and direction of transcription (arrows) of sense mRNA and antisense lncRNA molecules are indicated. B) SINEUP ruling effect is to enhance translation of partially overlapping sense protein-coding mRNAs without affecting the expression levels of the target mRNA.

Our group has recently discovered and characterized a new family of antisense lncRNAs whose ruling effect is to promote translation of partially overlapping sense protein-coding mRNAs without affecting the expression levels of the target mRNA [96], [97]. These molecules have been named SINEUPs, as an embedded inverted SINE B2 element is required to UP-regulate translation. SINEUP translation enhancement activity has been referred to also as gene-specific “knock-up”. SINEUP activity depends on two functional domains (Fig. 3):

• the “Binding Domain” (BD), a sequence at the 5′ of SINEUP lncRNA, that overlaps in opposite orientation to the target coding mRNA; it confers target specificity.
• the “Effector Domain” (ED), a downstream-embedded inverted SINE B2 element in the non-overlapping portion of SINEUP lncRNA; it functions as activator of translation.

SINEUP molecules act by selecting target mRNAs through their BD and by triggering enhanced loading to heavy polysomes for more efficient translation *via* their ED. Indeed, removal of the overlapping sequence or the SINE B2 repeat completely abrogates the translational up-regulation capabilities of SINEUPs [96]. Therefore, SINEUPs are modular antisense lncRNAs, in which the combined activity of the two domains (BD and ED) confers gene-specific translation enhancement effects. As such, BD can be designed to redirect translation up-regulation activity to potentially any target gene of interest. Gene-specific BDs are typically designed around the initiating AUG codon and overlapping part of the 5′ untranslated sequence and a portion of the coding sequence [98]. Despite the exact rules governing sense mRNA and SINEUP interaction are presently not known, increasing number of examples suggest a certain degree of flexibility in BD design (unpublished data). Proof-of-principle was originally provided by the design a synthetic SINEUP to knock-up GFP. As predicted, SINEUP-GFP increased GFP protein quantities without affecting its mRNA levels [96]. SINEUP-mediated knock-up of overexpressed proteins is typically in the range of 2-to-5-fold [96], [97] and more evident for difficult-to-express proteins (unpublished data). This seems to be true for endogenous genes as well as for overexpressed proteins. Presently we do not know the exact mechanism(s) regulating SINEUP activity. We can envision that different cellular systems controlling protein homeostasis may at the end impact the overall efficacy of SINEUP-mediated knock-up effects. Given their modular structure and their ability to target mRNA for more efficient translation, synthetic SINEUPs have been recently tested as an innovative tool to treat conditions of reduced gene dosage. In our recent work, we designed synthetic SINEUPs to target endogenous DJ-1 mRNA, a gene involved in recessive familial forms of Parkinson's Disease, and we could knock-up endogenous DJ-1 protein levels up to 3-fold in 3 different neuronal cell lines *in vitro*[97]. Subsequently, in a collaborative effort aimed at proving that SINEUP technology can also be applied *in vivo*, we could rescue the defective gene expression in a medakafish model of Microphtalmia with Linear Skin Lesion, a human disorder characterized by haploinsufficient dosage of COX7b protein [99].

A large number of incurable diseases are caused by a haploinsufficient dosage of a relevant gene. Classical chemical screenings are currently employed to identify small-molecule compounds that may modulate mRNA stability and/or translatability, by targeting control sequence elements or accessory proteins [100], [101]. Nucleic acid-based drugs represent an alternative approach to treat such disorders. While a number of small- and micro-RNAs are designed to promote transcription [102], [103], SINEUPs provide gene-specific up-regulation at a post-transcriptional level.

As gene-specific enhancers of translation, SINEUPs could represent an attractive molecular tool to implement the pipelines of recombinant protein production. Important issues need to be taken into account for the use of synthetic SINEUPs in biomanufacturing: 1) SINEUPs need to be active in mammalian cell lines used for the production of recombinant proteins in biomanufacturing pipelines; 2) SINEUPs need to be scalable to target potentially any protein of interest; 3) SINEUPs need to be effective for secreted proteins.

First, the versatility of synthetic SINEUPs was tested using SINEUP-GFP in mammalian cells *in vitro*. More than 10 different cell lines of human, monkey, mouse and hamster origin were tested and proved effective to support SINEUP-mediated knock-up [97], [104]. More importantly, GFP up-regulation was observed in mammalian cell factories, as in HEK293 and in suspension culture of CHO cells [92], [97]. Subsequent work then demonstrated that synthetic SINEUPs could be engineered to target amino-terminal tags used in chimeric protein for production and purification. In addition to GFP [96], [97], this was also shown for FLAG [97] and HA [104]. A high-throughput automated fluorescence-based detection system has been recently set-up to screen large numbers of SINEUPs using GFP-fusion chimera (Takahashi H. et al., submitted; Takahashi H. and Kozhuharova A., personal communication).

Recombinant MAbs are one of the emerging classes of biopharmaceuticals with important therapeutic applications. Most mAbs are produced at large scale as secreted proteins in CHO cells grown in suspension. A proof-of-principle study showed that synthetic SINEUP, targeting a secreted version of Luciferase reporter gene, could efficiently knock-up its quantities acting at the post-transcriptional level [92]. Moreover, SINEUPs could be exploited with success to increase the expression of secreted proteins targeting different leader peptides (interleukin-6, mouse immunoglobulins, elastin) [92]. SINEUPs were also used to increase the production of a recombinant anti-HIV antibody, further supporting the versatility of the technology [104].

Altogether, SINEUPs represent a versatile molecular tool to increase the synthesis of recombinant proteins at a small-, medium- and large-scale production. Among the approaches to improve translation mentioned in this review, SINEUP is peculiar in that it is not based on the optimization of the target mRNA sequence.

Therefore, this tool will not compete with the other existing methods currently used to increase protein yields, but it can be used in addition to them.

## 3 Summary and Outlook

Many gene features are important to achieve high levels in the synthesis of recombinant proteins. The advent of powerful bioinformatics techniques in the past decade has generated a bunch of information on the regulation of protein translation. We no longer see translational regulation as an intricate mechanism rather we can look inside it and rationally intervene on mRNA sequence and structure with the aim to maximize its translatability.

With SINEUP lncRNAs, we added a new tool to increase translation of proteins that, at least to our best knowledge, acts independently from the target mRNA structure. Still we do not know the exact rules governing SINEUP activity, therefore SINEUP molecules are currently empirically designed and tested. However, we could reasonably expect that, as for other RNA-based mechanisms (RNAi, for example) [105], SINEUP comply specific rules linking RNA sequence to function and that could be easily included in an algorithm for the *in silico* design of SINEUP molecules.

With system biology tools becoming increasingly accessible, we will finally be able to develop a clear understanding of cell regulation and therefore discover the rational basis for cell engineering. At that point, SINEUP technology will achieve its true potential.

## Conflict of Interest

SZ and SG declare competing financial interests as co-founders and members of TransSINE Technologies (www.transsine.com). SG and SZ are named inventors in patent issued in the US Patent and Trademark Office on SINEUPs and licensed to TransSINE Technologies. DC, LP and FP declare no competing financial interests.

## Figures and Tables

**Table 1:** Some example of tools freely available online to predict/design RNA secondary structure, codon optimization and Signal peptide design.

RNA secondary structure prediction and optimization		
Tool	Web page	Ref.
ViennaRNA web service	http://rna.tbi.univie.ac.at	[106]
RNAsoft	http://www.rnasoft.ca/	[107]
Freiburg RNA tools	http://rna.informatik.uni-freiburg.de/	[108]
mRNA optimizer	http://bioinformatics.ua.pt/software/mrna-optimiser/	[109]
CoFold	http://www.e-rna.org/cofold/	[110]
RNA structure package	https://github.com/lulab/RME	[111]


Codon optimization		
Tool	Web page	Ref.
OptimumGene	http://www.genscript.com/	
Gene Designer	https://www.dna20.com/	[112]
Codon Optimization Tool	https://eu.idtdna.com/	
Codon Adaptation Tool	http://www.jcat.de	[113]
Optimizer	http://genomes.urv.es/OPTIMIZER/	[114]
Codon Optimization OnLine	http://cool.syncti.org/	[115]
Gene Optimizer	https://www.thermofisher.com/	[68], [116]
Visual Gene Developer	http://visualgenedeveloper.net/	[117]
COStar	http://life.sysu.edu.cn/COStar/COStar.html/	[118]
EUGene	http://bioinformatics.ua.pt/eugene	[119]

*Signal peptide optimization*		
UTR-Tailortech	http://www.unitargeting.com/tools.html	[84]
